# TREM-1 Expression on the Surface of Neutrophils in Patients With Visceral Leishmaniasis Is Associated With Immunopathogenesis

**DOI:** 10.3389/fcimb.2022.863986

**Published:** 2022-03-24

**Authors:** Lays Gisele Santos Bomfim, Lucas Sousa Magalhães, Lorrany Santana Rodrigues, Aline Silva Barreto, Camilla Natália Oliveira Santos, Priscila Lima dos Santos, Cristiane Bani Corrêa, Kiyoshi Ferreira Fukutani, Dalmo Correia Filho, Angela Maria da Silva, Michael Wheeler Lipscomb, Tatiana Rodrigues de Moura

**Affiliations:** ^1^ Health Sciences Graduate Program, Federal University of Sergipe, Aracaju, Brazil; ^2^ Laboratory of Molecular Biology and Immunology, Federal University of Sergipe, Aracaju, Brazil; ^3^ Department of Health Education, Federal University of Sergipe, Lagarto, Brazil; ^4^ Department of Morphology, Federal University of Sergipe, São Cristóvão, Brazil; ^5^ Instituto Gonçalo Moniz, Fundação Oswaldo Cruz (FIOCRUZ), Salvador-Bahia, Brazil; ^6^ Department of Biology, Howard University, Washington, DC, United States

**Keywords:** visceral leishmaniasis (VL), *Leishmania infantum*, neutrophils, TREM-1, inflammation

## Abstract

Visceral leishmaniasis (VL) is a systemic chronic and potentially fatal disease for humans. Mechanisms related to the dysregulation of the inflammatory response may be involved in both the pathogenesis and prognosis of VL. Triggering Receptor Expressed on Myeloid Cells-1 (TREM-1) is a receptor constitutively expressed on neutrophils and monocyte subsets. The protein serves to regulate and amplify inflammatory responses. This study aimed to evaluate the expression profile of TREM-1 on the surface of neutrophils from patients with VL at varying time points during leishmanicidal treatment. For this purpose, neutrophils were isolated from the peripheral blood of patients with VL at different stages of treatment, which include 0, 7, and 30 days after treatment. Surface TREM-1 expression was assessed by immunophenotyping neutrophil populations. In addition, the association of TREM-1 expression on the surface of neutrophils with clinical and laboratory parameters and serum levels of inflammatory mediators was also evaluated. Results demonstrate a lower surface expression of TREM-1 in VL patients in the absence of treatment. However, increased levels of TREM-1 expression were observed 7 and 30 days after the start of treatment, with levels similar to those of healthy controls. TREM-1 expression was directly correlated with lymphocyte and erythrocyte count and indirectly correlated with spleen and liver size. Furthermore, elevated levels of TREM-1 expression were also correlated with lower serum levels of interleukin (IL)-22. Taken together, these results suggest that infection by *Leishmania infantum* leads to depressed TREM-1 expression on the neutrophil surface and may contribute to the inflammatory imbalance that characterizes active VL disease.

## Introduction

Visceral leishmaniasis (VL) is a systemic chronic and potentially fatal disease for humans. Typical of countries that are located in parts of the tropical and subtropical regions, VL is classified as a neglected tropical disease caused by the intracellular protozoa of the genus *Leishmania* (Burza et al., 2018). It is estimated that 50,000–90,000 new cases occur worldwide annually, with only 25%–45% reported to the World Health Organization (WHO). *Leishmania infantum* is the species most commonly involved in causing VL (Alvar et al., 2004; Dantas-Torres, 2006), with its presence endemic in 12 countries (Pan American Health Organization, 2019).

The parasite *L. infantum* is transmitted during the blood meal of infected female sand flies of the genus *Lutzomyia.* After infection, the parasite migrates from the skin to internal organs such as the spleen, liver, and bone marrow. Most individuals infected with the parasites are able to well control the infection, resulting in asymptomatic or subclinical diagnoses. However, some develop symptomatic disease resulting in high fever, weight loss, anemia, leukopenia, and hepatosplenomegaly. Furthermore, the presentation of the clinical form of the disease is associated with the virulence of the parasite strain, immune competency of the host, and other nutritional and genetic factors (Boakye et al., 2005). Importantly, the infection can be fatal in clinical symptomatic individuals if left untreated (Murray et al., 2005).

Neutrophils are the first cells to be recruited to the site of infection during VL. As one of the principal mediators of inflammation, neutrophils can eliminate pathogens through several mechanisms, which include phagocytosis, production of toxic molecules such as reactive oxygen species (ROS), defensins, release of enzymes, and neutrophil extracellular traps (NETs) (Mantovani et al., 2011; Burn et al., 2021). These immune cells also play a crucial role in the resolution of inflammation, secretion of cytokines, and production of pro-resolution mediators (Mantovani et al., 2011; Burn et al., 2021).

Innate immunity is initiated with the recognition of various pathogen-associated microbial patterns (PAMPs) by pattern recognition receptors (PRRs) such as Toll-like receptors (TLRs) (Akira et al., 2006). Neutrophils recognize the presence of PAMPs through PRRs and activate signal transduction pathways to promote pro-inflammatory events and release key inflammatory cytokines such as tumor necrosis factor (TNF)-α, interleukin (IL)-1β, IL-8, macrophage inflammatory protein (MIP)-1α, and MIP-1β (van Zandbergen et al., 2002; Mantovani et al., 2011; Burn et al., 2021). Activation of neutrophils is an important step in controlling infections, as the process can serve as a feedforward mechanism to recruit the inflammatory state, recruit more innate immune cells to the site of inflammation, and contribute to the immunomodulation of the adaptive immune response (Fortin et al., 2006). However, in severe VL cases, appropriate immune responses are curtailed, leading to an inability to adequately resolve the infection resulting in chronic immune responses that then lead to excessive collateral tissue damage (dos Santos et al., 2016; Samant et al., 2021).

Triggering Receptor Expressed on Myeloid Cells-1 (TREM-1) is a member of the immunoglobulin superfamily expressed on the cell surface of neutrophil, monocyte, and macrophage myeloid subsets (Bouchon et al., 2000). Expression of the gene in these immune cell subsets is regulated by recognition of microbial products. Furthermore, activation and expression of this receptor occur in tandem with TLR signaling, with the help of an adapter protein DNAX activation protein of 12kDa (DAP12), resulting in the production of a variety of pro-inflammatory cytokines that amplify the innate and adaptive immune responses (Arts et al., 2013). TREM-1 can also be produced in its soluble form (sTREM-1). Notably, high serum levels of this soluble form are indicative of microbial infections and non-infectious inflammatory diseases (Gibot et al., 2004; Molad et al., 2015).

Previous work by our laboratory group demonstrated that high levels of circulating sTREM-1 correlates directly with VL disease severity. Moreover, we found that neutrophils from healthy donors *in vitro* infected with *L. infantum* increase TREM-1, DAP12, and IL-8 gene expression, while increasing the presence of sTREM-1 (Bomfim et al., 2017). However, the role of the membrane-bound TREM-1 receptor is still poorly understood in the context of VL. Therefore, the present study investigated the membrane-bound expression profile of TREM-1 in neutrophils from patients with VL in different time points of leishmanicidal treatment. Lastly, the relationship of TREM-1 with clinical and laboratory parameters and serum levels of inflammatory mediators was investigated in patients with VL.

## Materials and Methods

### Ethics Statement

This study was approved by the Ethics Committee of the University Hospital of the Federal University of Sergipe (CAAE-53366916.8.0000.5546 and CAAE-97770318.0.0000.5546). All clinical investigations were conducted with informed consent obtained from all participants or legal guardians in accordance with the Declaration of Helsinki.

### Subjects and Sample Collection

This study was performed with healthy human donors without infectious or other inflammatory diseases (experiment flow cytometry: n = 8; cytokine serum levels: n = 12) and with patients diagnosed with VL (n = 7) recruited at the Reference Center at the University Hospital in Sergipe, Brazil. The clinical criteria used for inclusion of patients with VL were clinical symptoms and signs, such as fever, weight loss, anemia, enlargement of spleen and liver, pancytopenia, and hypergammaglobulinemia. VL diagnosis was confirmed by direct observation of *Leishmania* in bone marrow aspirates or positive culture in Novy, Mac Neal and Nicole (NNN) media (Sigma-Aldrich, St. Louis, MO, USA) or positive rK39 serological test (KalazarDetect^®^ Rapid Test: InBios International Inc., Seattle, WA, USA). Patients underwent standard VL treatment, and blood was collected from the patient three times: before chemotherapy for leishmaniasis (pretreatment, D0) and 7 days (D7) and 30 days (D30) after treatment initiation. Pregnant women, patients receiving immunosuppressive treatments, and patients with comorbidities such as diabetes, HIV, Human T-lymphotropic virus 1 (HTLV-1), and malignancy, were excluded.

### Neutrophil Isolation and TREM-1 Detection on the Neutrophil Surface by Flow Cytometry

Neutrophils were isolated from heparinized peripheral blood by density gradient centrifugation using Histopaque 1077 (Sigma, St. Louis, MO, USA) and centrifuged at room temperature for 30 min at 400 × g. The plasma and cloud containing the mononuclear cells were removed from the top layer. The cloud containing the neutrophils (>95% of the cells) was collected, and contaminant red blood cells were removed by hypotonic lysis. Next, neutrophils were washed with Phosphate-buffered saline (PBS) and resuspended in Roswell Park Memorial Institute (RPMI) 1640 media (Gibco, USA) supplemented with 1% nutridoma (Roche, Indianapolis, IN, USA) and 1% penicillin/streptomycin.

To evaluate the TREM-1 surface expression on neutrophils, the cells (2.5 × 10^6^ cells/ml) were incubated with 100 μl of blocking serum (2% fetal goat and 2% fetal bovine calf sera) for 20 min at 4°C to inhibit non-specific binding through FcγRs. Next, the cells were stained with CD11b-APC and anti-CD354/TREM-1-PE antibodies (BioLegend) for 20 min at 4°C and then washed and resuspended in PBS. A minimum of 30,000 events were acquired using the FACS CANTO II (BD Biosciences) and analyzed using FlowJo v10.0 software (Tree Star). For multiparameter cytometry analysis, the neutrophil population was selected using the size (Forward Scatter - FSC) and granularity (Side Scatter - SSC) parameters, into that gating was selected the CD11b+ cells, and into that gate were analyzed the expression of TREM-1+ cells. CD11b was used as a neutrophil marker (Lakschevitz et al., 2016).

### Quantification of Inflammatory Mediators in Serum

Inflammatory mediators were measured in the serum (previously stored at -−80°C), that was obtained from peripheral blood of patients with VL and healthy controls (HCs). Determination of sTREM-1 concentrations was performed by specific enzyme-linked immunosorbent assay (ELISA) kit (DuoSet, R&D Systems, Abingdon, UK). The absorbance at 450 nm was measured using a microplate reader (Epoch, BioTek, Luzern, Switzerland) with a wavelength correction set at 570 nm to subtract background. A standard curve was generated for each set of samples assayed using the manufacturer’s recommended protocol.

The cytokines IL-4, IL-5, IL-6, IL-12p70, IL-17A, IL-22, TNF-α, and interferon (IFN)-γ was done by multiplex assay (ProcartaPlex Multiplex Immunoassay–Human Custom, Thermo, Waltham, MA, USA), and the cytokine concentrations were analyzed by MILLIPLEX Analist 5.1 software (Merck Millipore, Billerica, USA).

### Statistical Analysis

Descriptive and statistical data analyses were performed. Kolmogorov–Smirnov normality test was applied. Differences between the groups were calculated using Student’s t test or one-way ANOVA for parametric data. Mann–Whitney test or Kruskal–Wallis test with the Dunn’s multiple comparisons test were used for non-parametric data. Correlation analysis was performed using Spearman correlation test. Differences with p < 0.05 were considered statistically significant. Analyses were performed using GraphPad Prism 8.0.2 software. Heatmaps were made to represent the expression of the TREM-1 marker through the signal intensity, as previously described (Gu et al., 2016). Unsupervised two-way hierarchical cluster analysis (Ward’s method) was utilized to test whether VL patients at different time points of leishmanicidal treatment and endemic HCs could be grouped separately based on the overall expression profile of serum markers.

## Results

### Demographic, Clinical, and Laboratorial Data of Patients

All patients had a diagnosis of VL confirmed by rK39 serology and/or parasite bone marrow culture. Clinical and laboratory parameters of patients with VL before and after leishmanicidal treatment were compared (**Table 1**). Patients with VL included in the study were composed of 43% women (n = 3) and 57% men (n = 4), with a mean age of 41 ± 15 years (mean ± SD). No significant differences in spleen or liver size were detected among cohorts over the course of treatment. As expected, it was observed that patients before starting treatment (D0) have anemia, thrombocytopenia, leukopenia coupled with significant neutropenia, and lymphopenia. Elevated levels of Aspartate transaminase (AST) enzyme were also observed in patients at time D0. On day 30 posttreatment (D30), all treated patients showed signs of recovery from the clinical symptoms (**Table 1**).

**Table 1:** Clinical and laboratory data of patients with VL at different times of leishmanicidal treatment.

*Parameters*	*D0*	*D7*	*D30*	*p-value*	*Posttest result*
Number of patients		7		–	–
Gender (M/F)		4/3		–	–
Age		41.00 ± 15.07		–	–
Spleen (cm)	6.29 ± 5.50	3.25 ± 4.47	0.4 ± 0.89	0.0110	n.s.
Liver (cm)	3.5 ± 2.75	0.83 ± 1.60	0.92 ± 1.63	0.524	n.s.
Hemoglobin (g/dl)	9.30 ± 1.92	9.49 ± 1.92	11.91 ± 0.66	0.0199	*
Hematocrit (%)	27.97 ± 5.75	28.95 ± 5.86	35.53 ± 1.90	0.0298	*
Erythrocytes (10^6^/mm³)	3.63 ± 0.89	3.68 ± 0.70	4.35 ± 0.27	0.0827	n.s.
Platelets (/mm³)	85,329 ± 29,969	166,486 ± 94,923	186,286 ± 65,304	0.0329	*
Leucocytes (/mm³)	2,459 ± 788.9	3,647 ± 1,375	6,009 ± 1,409	0.0001	*
Neutrophils (/mm³)	1,105 ± 662.9	1,898 ± 1,077	2,709 ± 685.3	0.0051	*
Lymphocytes (/mm³)	989.0 ± 267.5	1,298 ± 382.8	2,275 ± 699.8	0.0004	*, $
Eosinophils (/mm³)	42.29 ± 43.81	65.86 ± 77.87	561.1 ± 610.7	0.0047	*, $
Basophils (/mm³)	15.14 ± 16.97	109.9 ± 193.1	110.7 ± 159.6	0.1702	n.s.
Monocytes (/mm³)	281.6 ± 142.8	267.1 ± 197.6	354.0 ± 185.1	0.5094	n.s.
Urea (mg/dl)	26.85 ± 28.38	37.67 ± 21.00	32.67 ± 11.85	0.3115	n.s.
Creatinine (mg/dl)	0.77 ± 0.11	1.10 ± 0.25	0.77 ± 0.12	0.0195	#
AST (U/L)	118.4 ± 112.1	81.29 ± 74.70	32.14 ± 13.68	0.0463	*
ALT (U/L)	80.29 ± 64.31	90.29 ± 63.78	37.29 ± 16.90	0.0410	n.s.

Values express the mean ± standard deviation. Data were analyzed using Kruskal–Wallis test with Dunn’s multiple comparisons posttest. Column with p-values represent the Kruskal–Wallis test. p-values from posttest are represented by the following: *p < 0.05 in D0 vs. D30; #p < 0.05 in D0 vs. D7; $p < 0.05 in D7 vs. D30; n.s., nonsignificant; AST, Aspartate transaminase; ALT, alanine aminotransferase.

The most common symptoms presented by the patients were fever and weight loss. The duration of disease before diagnosis ranged from 20 to 270 days, with an average of 103 days. Most patients (71.44%) underwent treatment with liposomal amphotericin B, with 2 patients (28.56%) treated with pentavalent antimonials.

### TREM-1 Expression in Neutrophils From Patients With Visceral Leishmaniasis at Different Time Points of Leishmanicidal Treatment

**Figure 1:**
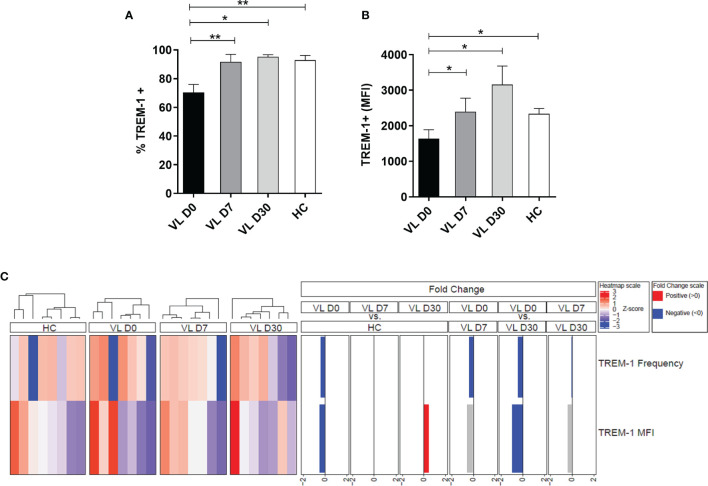
Expression of surface TREM-1 in neutrophils from visceral leishmaniasis (VL) patients at different times of leishmanicidal treatment and healthy controls (HCs). Neutrophils from patients with VL (n = 8) and healthy donors (n = 8) were isolated from the peripheral blood, and **(A)** the frequency and **(B)** MFI of neutrophils expressing TREM-1 were assessed by flow cytometry. Bars represent the mean ± SEM. Mann–Whitney test or Student’s t test were used to compare patients’ treatment times (VL D0, VL D7, and VL 30) with the HC, and the Friedman test with Dunn’s posttest or one-way ANOVA were used for paired analysis of patients in treatment times (VL D0, VL D7, and VL D30). *p < 0.05; **p < 0.01. **(C)** Cluster analysis and heatmap expressing the frequency and MFI of TREM-1 in the neutrophils of patients with VL at different times of treatment and in HCs. Fold changes were calculated, and statistically significant differences are highlighted in blue and red.

The surface expression of TREM-1 on neutrophils was measured in patients with VL at different time points of leishmanicidal treatment: before (pretreatment, D0), during (D7), and after the start of treatment (D30). Results revealed a lower frequency of TREM-1 expression on neutrophils of patients before treatment (D0) compared to that of HCs (mean ± SD: 70.39 ± 14.63 vs. 92.88 ± 9.22, respectively; p = 0.0059) (**Figure 1A**). The mean fluorescence intensity (MFI) was used to demonstrate increased or decreased overall levels of the receptor on the surface of neutrophils. Notably, lower TREM-1 MFI on neutrophils from pretreated VL patients (D0) was observed compared to that of HCs (mean ± SD: 1,643 ± 652.9 vs. 2,334 ± 431.5, respectively; p = 0.0292) (**Figure 1B**). When performing a paired analysis by comparing the expression and TREM-1 MFI of patients at different times of treatment, a difference was observed in relation to the frequency of TREM-1 between the D0 and D7 (p = 0.0099) and D0 and D30 (p = 0.0226) time points. The TREM-1 MFI was also significantly different between D0 and D7 (p = 0.0313) and D0 and D30 (p = 0.0469). These results demonstrate that in the days after the start of treatment (D7 and D30), the expression of the TREM-1 receptor in the patients’ neutrophils returned to levels similar to those found in healthy groups.

Subsequently, a heatmap was constructed to display the expression intensity values of TREM-1 utilizing fold change values of ≤2 and ≥-2 (**Figure 1C**). A hierarchical cluster analysis (Ward’s method) was used to describe the expression profile of TREM-1 in neutrophils from patients with VL at different times of treatment. Patients before treatment have a lower frequency and MFI of TREM-1 compared to HCs. This reduction in TREM-1 frequency was also observed among patients at time D0 compared to patients at 7 and 30 days after treatment. After 30 days of treatment, an increase in the frequency and MFI of TREM-1 was also observed on neutrophils in VL-treated cohorts.

**Figure 2:**
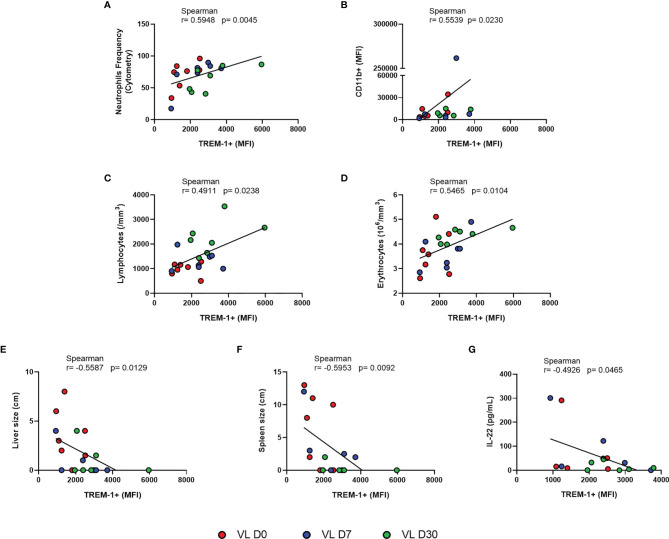
Correlation between MFI of TREM-1 and **(A)** neutrophil frequency, **(B)** MFI CD11b, and **(C–G)** laboratory parameters and clinical and IL-22 serum levels from patients with VL at different times of leishmanicidal treatment. Spearman correlation test. Patients with VL before treatment are represent in red; and after treatment initiation with 7 days in blue, and 30 days in green.

Since MFI is a measure that infers the fluorescence intensity of the antibody used and it is proportionally associated with the amount of molecules present on the cell surface, we used this measure to perform correlation analyses (Hogg et al., 2015). A strongly positive correlation was observed between TREM-1 MFI and the frequency of neutrophils (r = 0.5948, p = 0.0045) (**Figure 2A**) and with the CD11b MFI (r = 0.5539, p = 0.0230) (**Figure 2B**), which is a marker for myeloid subsets.

We then evaluated how TREM-1 MFI related to patients’ clinical profile. Results revealed that the TREM-1 MFI was positively correlated with lymphocyte (r = 0.4911, p = 0.0238) and erythrocyte (r = 0.5465, p = 0.0104) counts (**Figures 2C, D** **)**. A negative correlation was observed in the TREM-1 MFI with the sizes of the spleen (r = -0.5953, p = 0.0092) and liver (r = -0.5587, p = 0.0129) (**Figures 2E, F** **)**.

### Inflammatory Mediator Profile in Visceral Leishmaniasis Patients

Since VL is characterized by an intense circulation of several cytokines, investigations next evaluated the serum levels of some key biomarker indices related to VL and whether these were correlated with the expression of TREM-1 on neutrophils. We measured IL-4, IL-5, IL-6, IL-12p70, IL-17A, IL-22, TNF-α, and IFN-γ cytokine concentrations in the serum of VL patients and HCs. We found higher levels of IL-4, IL-6, IL-12p70, IL-17A, IL-22, IFN-γ, and sTREM-1 in patients before treatment (D0) when compared to those of HCs (**Table 2**). No significant differences were observed between D0 and HC for TNF-α or IL-5. After the initiation of leishmanicidal treatment, it was clearly observed that the general expression profile in VL patients at day 30 became similar to that observed in HCs, except for the mediators IL-4, IL-6, and sTREM-1, which still showed significant differences compared to HCs (**Table 2**).

**Table 2:** Inflammatory mediator profile of patients with VL at different times of leishmanicidal treatment.

*Inflammatory mediators (pg/ml)*	*D0*	*D7*	*D30*	*HC*	*Mann–Whitney test*	*Dunn’s posttest*
TNF-α	15.13 ± 13.22	5.55 ± 6.87	5.67 ± 6.60	4.13 ± 7.03	n.s.	n.s.
IL-4	15.59 ± 24.44	65.78 ± 107.9	5.44 ± 7.48	0.42 ± 1.36	*, #, $	n.s.
IL-5	44.44 ± 71.90	150.1 ± 256.2	15.46 ± 19.35	6.69 ± 9.89	#	n.s.
IL-6	41.73 ± 34.70	83.93 ± 97.43	12.33 ± 16.46	0.47 ± 0.91	*, #, $	+
IL-12p70	33.73 ± 44.39	128.5 ± 182.0	9.20 ± 13.10	2.51 ± 4.54	*, #	n.s.
IL-17A	8.26 ± 8.58	27.27 ± 37.91	2.65 ± 3.36	1.31 ± 3.17	*, #	n.s.
IL-22	74.56 ± 122.4	86.86 ± 113.0	15.79 ± 18.91	8.06 ± 21.54	*, #	n.s.
IFN-γ	8.77 ± 9.54	39.13 ± 78.74	1.99 ± 3.91	0.30 ± 0.98	*, #	n.s.
sTREM-1	86.61 ± 42.87	117.9 ± 60.12	85.50 ± 46.23	36.58 ± 33.88	*, #, $	n.s.

Values express the mean ± standard deviation. Mann–Whitney test was used to compare patients’ treatment times (VL D0, VL D7, and VL 30) with the healthy control (HC), and the Friedman test with Dunn’s posttest was used for paired analysis of patients in treatment times (VL D0, VL D7, and VL D30). Column with Mann–Whitney test and Dunn’s posttest represents p-values and are represented by the following: *p < 0.05 in D0 vs. HC; #p < 0.05 in D7 vs. HC; $p < 0.05 in D30 vs. HC; +p < 0.05 in D7 vs. D30; n.s., nonsignificant.

We then evaluated how TREM-1 MFI related to these inflammatory mediators within the serum. TREM-1 MFI was negatively correlated with IL-22 (r = -0.4926, p = 0.0465) (**Figure 2G**); no other cytokine had direct or indirect correlations. Furthermore, as IL-22 increases the production of pro-inflammatory molecules (Boniface et al., 2005), we investigated whether there was a correlation between IL-22 and the serum levels of other inflammatory mediators. We observed a positive correlation between IL-22 and IL-4, IL-5, IL-6, IL-12p70, IL-17A, and TNF-α. There was no correlation of IL-22 with sTREM-1 nor IFN-γ (**Supplementary Table S1**).

**Figure 3:**
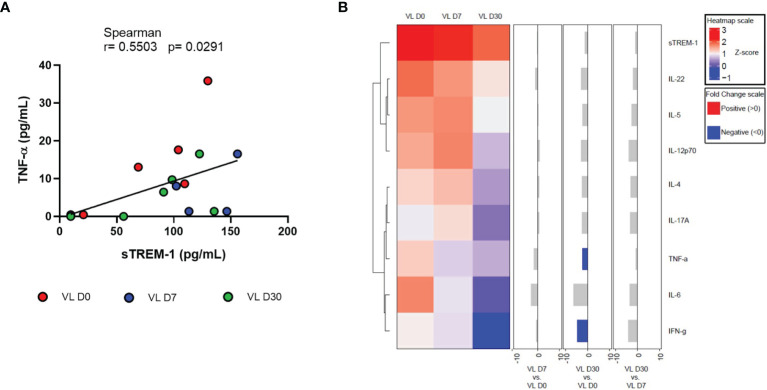
**(A)** Correlation between sTREM-1 with TNF-a serum levels from patients with VL at different times of leishmanicidal treatment. Spearman correlation test. **(B)** Cluster analysis and heatmap expressing serum inflammatory mediators in VL patients at different times of leishmanicidal treatment. Fold changes have been calculated, and significant differences are highlighted in blue.

We also assessed whether sTREM-1 levels in the patients’ serum could be associated with influencing cytokine levels. We observed a significant positive correlation with TNF-α serum level (r = 0.5503, p = 0.0291) (**Figure 3A**). There were no correlations of sTREM-1 with the clinicals and laboratorial paramenters of patients (liver and spleen size, hematological parameters and, liver and kidney enzyme levels).

Finally, results of the quantification of serum inflammatory mediators were used to perform the heatmap analysis (**Figure 3B**). In the hierarchical cluster analysis, the formation of two large clusters was observed: sTREM-1, IL-22, IL-5, and IL-12p70 and another with IL-4, IL-17A, TNF-α, IL-6, and IFN-γ. A negative fold change was observed, with a significant difference only with TNF-α and IFN-γ between times D30 and D0.

## Discussion

In the present study, we describe for the first time that low expression of the TREM-1 receptor on the surface of neutrophils is present in patients with VL. Upon anti-leishmaniasis treatment regimens, TREM-1 receptor expression returned to levels similar to those found in HCs. Ruiz-Pacheco et al. (2014) also had similar findings upon dengue infections; the authors observed a significant decrease in TREM-1 expression in the patients’ neutrophils during infection. Here, we observed that TREM-1 levels on the surface of circulating neutrophils appear to increase as the infection waned. Studies showed that a large proportion of the neutrophils in the blood of VL patients are immature cells (Yizengaw et al., 2016; Sharma et al., 2016). We hypothesized that a lower expression of the TREM-1 receptor in the neutrophil membrane of patients with VL before treatment may be associated with the release of premature neutrophils (with low expression of TREM-1) from the bone marrow to the systemic circulation. This hypothesis corroborates with the finding of Gingras et al. (2002), who showed that TREM-1 expression was associated with the maturation stage of myeloid cells. However, further studies are needed to investigate the bone marrow environment during *Leishmania* infection.

We found that the strong positive correlation between TREM-1 MFI and neutrophil frequency proves that neutrophils are a source of TREM-1 in VL. The low expression of TREM-1 in neutrophils was associated with clinical parameters of severity and bone marrow functioning, as for example, reduced levels of circulating lymphocytes and erythrocytes, and enlargement of the liver and spleen. As treatment progressed, improvement in these parameters was observed, associated with an increase in neutrophil TREM-1 expression. It is possible that *L. infantum* infection in the bone marrow compromises the expression of this receptor. A neutrophil with low TREM-1 expression may not perform its function correctly and thus favor the spread of the parasite and secondary infections.

Our previously published reports show that neutrophils from healthy donors infected with *L. infantum* have a lower expression of TREM-1 on the surface and higher concentrations of sTREM-1 in the supernatant (Bomfim et al., 2017). In particular, high levels of circulating sTREM-1 in patients were correlated directly with VL disease severity, with the highest levels of sTREM-1 observed in non-survivor VL patients (Bomfim et al., 2017). A greater reduction of TREM-1 on the surface of neutrophils and a significant increase in plasma levels of sTREM-1 also were observed in septic patients (Oku et al., 2013) and in patients with acute dengue infection (Ruiz-Pacheco et al., 2014).

Systemic inflammation has been described as a cause of clinical manifestations in leishmaniasis (Goto and Prianti, 2009). In VL, this inflammatory response is marked by the release of multiple cytokines (Peruhype-Magalhaes et al., 2005; Costa et al., 2012; Costa et al., 2013; dos Santos et al., 2016). Based on the finding of altered TREM-1 expression on neutrophils from VL patients, we investigated the relationship of TREM-1 expression in neutrophils with serum levels of inflammatory mediators in VL patients. Results revealed high levels of cytokines such as IL-4, IL-6, IL-12p70, IL-17A, IL-22, IFN-γ, and sTREM-1 before treatment, which after antiparasitic treatment returned to values similar to those found in HCs, corroborating data present in the literature (dos Santos et al., 2016; Araújo-Santos et al., 2017).

We found that the TREM-1 MFI was negatively correlated with IL-22 serum levels. IL-22 is a poorly studied cytokine; some studies show that it may be associated with skin and mucosal immunity against infections, but when produced at high levels or in the context of other pro-inflammatory cytokines, it promotes an increase in pathologies (Sonnenberg et al., 2010). Boniface et al. (2005) reported that IL-22 increases the production of pro-inflammatory molecules (Boniface et al., 2005). An interesting finding in the present study is that it was observed that the amount of IL-22 presented a positive correlation with the serum levels of the inflammatory mediators IL-4, IL-5, IL-6, IL-12p70, IL-17A, and TNF- α but no correlation with sTREM-1 and IFN-γ. In *L. donovani* infection, it was observed that IL-17 and IL-22 lead to protection against VL, and it is suggested that these cytokines act by complementing the protective role of Th1 cytokines in a non-dependent way (Pitta et al., 2009).

Previous studies have demonstrated an association between the decrease on the TREM-1 surface of neutrophils and the increase in serum levels of sTREM-1 (Oku et al., 2013). But here it was not possible perhaps because of the small sample size or because critically ill patients were not evaluated, as has been reported in previous studies (Oku et al., 2013; Bomfim et al., 2017). Here, we showed that sTREM-1 concentration was positively correlated with TNF-α levels. In neonatal sepsis, a positive correlation was observed between sTREM-1 and TNF-α and other pro-inflammatory cytokines such as IL-6 and IL-8 (Qian et al., 2014). In contrast, when leukocytes were treated with the LP17 peptide, which specifically aims to block TREM-1, the secretion of pro-inflammatory cytokines produced after exposure to *Escherichia coli* was attenuated (Qian et al., 2014). A study by van Bremen et al. (2013) showed that low TREM-1 expression in neutrophils may be associated with hyporesponsiveness during severe sepsis (van Bremen et al., 2013). The authors found correlations between TREM-1 expression in neutrophils and cytokine induction, suggesting the involvement of this receptor in the ability to produce cytokines.

The heatmap of inflammatory mediators at different treatment times showed a significant difference in the fold change with TNF-α and IFN-γ only, demonstrating that 30 days after the start of treatment, patients present these cytokines with reduced levels compared to the phase of pretreatment. IFN-γ is involved in parasite control (Kima and Soong, 2013). Several studies show that high levels of TNF-α have been independently implicated in VL-associated disease severity and death (Costa et al., 2013; dos Santos et al., 2016). Thus, once the patient progresses to clinical cure, a reduction in the serum levels of these cytokines is expected.

Taken together, these results suggest that infection by Leishamina infatum alters the expression of TREM-1 on the surface of neutrophils which leads to an imbalance in the regulation of inflammatory responses, which prevents adequate defense against VL disease. Furthermore, findings highlight the use of TREM-1 expression on the neutrophil surface as a biomarker for VL disease progression. Although the study was the first to assess all these biomarkers together as predictors of severity for VL, it has some limitations that should be noted, as the limited number of patients. More studies are needed for an increase in sample sizes. Studies by employing animal models to perform TREM-1 blockade and to assess the action of Leishmania in vivo, as well as, intracellular signaling and proteomic studies would also serve to elucidate the mechanistic role of the TREM-1 pathway in VL. Lastly, we also emphasize that other molecules and factors interfere in the immunopathogenesis of VL, where the combination of these can culminate in a protective or immunosuppressive response.

## Data Availability Statement

The original contributions presented in the study are included in the article/**Supplementary Material**. Further inquiries can be directed to the corresponding author.

## Ethics Statement

This study involved human participants and, therefore, was reviewed and approved by the Ethics Committee of the University Hospital of the Federal University of Sergipe (CAAE-53366916.8.0000.5546 and CAAE-97770318.0.0000.5546). Written informed consent to participate in this study was provided by all participating subjects or their legal guardians.

## Author Contributions

Conceived and designed the experiments: LB and TdM. Follow-up of patients: AS, LB, LM, LR, and AB. Performed the experiments: LB, LM, LR, AB, PL, and CS. Analyzed the data: LB, ML, CC, KF, AS, DC, and TdM. Contributed reagents/materials/analysis tools: ML and TdM. Wrote the article: LB, ML, and TdM. All authors contributed to the article and approved the submitted version.

## Funding

This work was supported by grants: The fellowships received by LB, LM, LR, and CS were financed by the following programs: Coordenação de Aperfeiçoamento de Pessoal de Nível Superior (CAPES); Fundação de Apoio à Pesquisa e à Inovação Tecnológica do Estado de Sergipe MS/CNPq/FAPITEC/SE/SES–N° 06/2018: 019.203.00933/2018-0 PPSUS-Sergipe (TRM); Conselho Nacional de Desenvolvimento Científico e Tecnológico MCTIC/CNPq N° 28/2018: 434623/2018-0 (TRM); National Institutes of Health, NIH Grant #SC1GM127207 (ML), Department of Defense, DOD Grant #W911NF-14-1-01123 (ML), and National Sciences Foundation, NSF Grant #1428768 (ML).

## Conflict of Interest

The authors declare that the research was conducted in the absence of any commercial or financial relationships that could be construed as a potential conflict of interest.

## Publisher’s Note

All claims expressed in this article are solely those of the authors and do not necessarily represent those of their affiliated organizations, or those of the publisher, the editors and the reviewers. Any product that may be evaluated in this article, or claim that may be made by its manufacturer, is not guaranteed or endorsed by the publisher.

## References

[B1] Akira S. Uematsu S. Takeuchi O. (2006). Pathogen Recognition and Innate Immunity. Cell 124 (4), 783–801. doi: 10.1016/j.cell.2006.02.015 16497588

[B2] Alvar J. Cañavate C. Molina R. Moreno J. Nieto J. (2004). Canine Leishmaniasis. Adv. Parasitol. 57, 1–88. doi: 10.1016/S0065-308X(04)57001-X 15504537

[B3] Araújo-Santos T. Andrade B. B. Gil-Santana L. Luz N. F. dos Santos P. L. de Oliveira F. A.. (2017). Anti-Parasite Therapy Drives Changes in Human Visceral Leishmaniasis-Associated Inflammatory Balance. Sci. Rep. 7 (1), 4334. doi: 10.1038/s41598-017-04595-8 28659627 PMC5489532

[B4] Arts R. J. W. Joosten L. A. B. van der Meer J. W. M. Netea M. G. (2013). TREM-1: Intracellular Signaling Pathways and Interaction With Pattern Recognition Receptors. J. Leukoc. Biol. 93 (2), 209–215. doi: 10.1189/jlb.0312145 23108097

[B5] Boakye D. A. Wilson M. D. Kweku M. (2005). A Review of Leishmaniasis in West Africa. Ghana Med. J. 39 (3), 94–97. 17299551 PMC1790817

[B6] Bomfim L. G. S. Magalhães L. S. Santos-Filho M. A. A. Peres N. T. A. Corrêa C. B. Tanajura D. M.. (2017). Leishmania Infantum Induces the Release of sTREM-1 in Visceral Leishmaniasis. Front. Microbiol. 8, 1–8. doi: 10.3389/fmicb.2017.02265 29201022 PMC5696592

[B7] Boniface K. Bernard F.-X. Garcia M. Gurney A. L. Lecron J.-C. Morel F. (2005). IL-22 Inhibits Epidermal Differentiation and Induces Proinflammatory Gene Expression and Migration of Human Keratinocytes. J. Immunol. 174 (6), 3695–3702. doi: 10.4049/jimmunol.174.6.3695 15749908

[B8] Bouchon A. Dietrich J. Colonna M. (2000). Cutting Edge: Inflammatory Responses Can Be Triggered by TREM-1, a Novel Receptor Expressed on Neutrophils and Monocytes. J. Immunol. 164 (10), 4991–4995. doi: 10.4049/jimmunol.164.10.4991 10799849

[B9] Burn G. L. Foti A. Marsman G. Patel D. F. Zychlinsky A. (2021). The Neutrophil. Immunity 54 (7), 1377–1391. doi: 10.1016/j.immuni.2021.06.006 34260886

[B10] Burza S. Croft S. L. Boelaert M. (2018). Leishmaniasis. Lancet 392 (10151), 951–970. doi: 10.1016/S0140-6736(18)31204-2 30126638

[B11] Costa A. S. A. Costa G. C. de Aquino D. M. C. de Mendonça V. R. R. Barral A. Barral-Netto M.. (2012). Cytokines and Visceral Leishmaniasis: A Comparison of Plasma Cytokine Profiles Between the Clinical Forms of Visceral Leishmaniasis. Mem. Inst. Oswaldo Cruz 107 (6), 735–739. doi: 10.1590/S0074-02762012000600005 22990961

[B12] Costa D. L. Rocha R. L. Carvalho R. M. A. Lima-Neto A. S. Harhay M. O. Costa C. H. N.. (2013). Serum Cytokines Associated With Severity and Complications of Kala-Azar. Pathog. Glob. Health 107 (2), 78–87. doi: 10.1179/2047773213Y.0000000078 23683334 PMC4001482

[B13] Dantas-Torres F. (2006). Leishmania Infantum Versus Leishmania Chagasi: Do Not Forget the Law of Priority. Mem. Inst. Oswaldo Cruz 101 (1), 117–118. doi: 10.1590/S0074-02762006000100024 16699722

[B14] dos Santos P. L. de Oliveira F. A. Santos M. L. B. Cunha L. C. S. Lino M. T. B. B. de Oliveira M. F. S. S.. (2016). The Severity of Visceral Leishmaniasis Correlates With Elevated Levels of Serum IL-6, IL-27 and Scd14. PloS Negl. Trop. Dis. 10 (1), 1–16. doi: 10.1371/journal.pntd.0004375 PMC4729473 26814478

[B15] Fortin C. F. Lesur O. Fulop T. (2006). Effects of TREM-1 Activation in Human Neutrophils: Activation of Signaling Pathways, Recruitment Into Lipid Rafts and Association With TLR4. Int. Immunol. 19 (1), 41–50. doi: 10.1093/intimm/dxl119 17098818

[B16] Gibot S. Kolopp-Sarda M.-N. Béné M.-C. Bollaert P. Lozniewski A. Mory F.. (2004). A Soluble Form of the Triggering Receptor Expressed on Myeloid Cells-1 Modulates the Inflammatory Response in Murine Sepsis. J. Exp. Med. 200 (11), 1419–1426. doi: 10.1084/jem.20040708 15557347 PMC2211948

[B17] Gingras M.-C. Lapillonne H. Margolin J. F. (2002). TREM-1, MDL-1, and DAP12 Expression Is Associated With a Mature Stage of Myeloid Development. Mol. Immunol. 38 (11), 817–824. doi: 10.1016/S0161-5890(02)00004-4 11922939

[B18] Goto H. Prianti M. D. G. (2009). Immunoactivation and Immunopathogeny During Active Visceral Leishmaniasis. Rev. Inst. Med. Trop. Sao Paulo 51 (5), 241–246. doi: 10.1590/S0036-46652009000500002 19893975

[B19] Gu Z. Eils R. Schlesner M. (2016). Complex Heatmaps Reveal Patterns and Correlations in Multidimensional Genomic Data. Bioinformatics 32 (18), 2847–2849. doi: 10.1093/bioinformatics/btw313 27207943

[B20] Hogg K. Thomas J. Ashford D. Cartwright J. Coldwell R. Weston D. J.. (2015). Quantification of Proteins by Flow Cytometry: Quantification of Human Hepatic Transporter P-Gp and OATP1B1 Using Flow Cytometry and Mass Spectrometry. Methods 82, 38–46. doi: 10.1016/j.ymeth.2015.03.030 25916617

[B21] Kima P. E. Soong L. (2013). Interferon Gamma in Leishmaniasis. Front. Immunol. 4, 1–5. doi: 10.3389/fimmu.2013.00156 23801993 PMC3685816

[B22] Lakschevitz F. S. Hassanpour S. Rubin A. Fine N. Sun C. Glogauer M. (2016). Identification of Neutrophil Surface Marker Changes in Health and Inflammation Using High-Throughput Screening Flow Cytometry. Exp. Cell Res. 342 (2), 200–209. doi: 10.1016/j.yexcr.2016.03.007 26970376

[B23] Mantovani A. Cassatella M. Costantini C. Jaillon S. (2011). Neutrophils in the Activation and Regulation of Innate and Adaptive Immunity. Nat. Rev. Immunol. 11 (8), 519–531. doi: 10.1038/nri3024 21785456

[B24] Molad Y. Ofer-Shiber S. Pokroy-Shapira E. Oren S. Shay-Aharoni H. Babai I. (2015). Soluble Triggering Receptor Expressed on Myeloid Cells-1 is a Biomarker of Anti-CCP-Positive, Early Rheumatoid Arthritis. Eur. J. Clin. Invest. 45 (6), 557–564. doi: 10.1111/eci.12442 25832796

[B25] Murray H. W. Flanders K. C. Donaldson D. D. Sypek J. P. Gotwals P. J. Liu J.. (2005). Antagonizing Deactivating Cytokines To Enhance Host Defense and Chemotherapy in Experimental Visceral Leishmaniasis. Infect. Immun. 73 (7), 3903–3911. doi: 10.1128/IAI.73.7.3903-3911.2005 15972476 PMC1168607

[B26] Oku R. Oda S. Nakada T. A. Sadahiro T. Nakamura M. Hirayama Y.. (2013). Differential Pattern of Cell-Surface and Soluble TREM-1 Between Sepsis and SIRS. Cytokine 61 (1), 112–117. doi: 10.1016/j.cyto.2012.09.003 23046618

[B27] Pan American Health Organization (2019). “Manual of Procedures for Surveillance and Control of Leishmaniasis in the Americas,” in World Health Organization, 166. (Washington, D.C.: PAHO).

[B28] Peruhype-Magalhaes V. Martins-Filho O. Prata A. de A. Silva L. Rabello A. Teixeira-Carvalho A.. (2005). Immune Response in Human Visceral Leishmaniasis: Analysis of the Correlation Between Innate Immunity Cytokine Profile and Disease Outcome. Scand. J. Immunol. 62 (5), 487–495. doi: 10.1111/j.1365-3083.2005.01686.x 16305646

[B29] Pitta M. G. R. Romano A. Cabantous S. Henri S. Hammad A. Kouriba B.. (2009). IL-17 and IL-22 Are Associated With Protection Against Human Kala Azar Caused by Leishmania Donovani. J. Clin. Invest. 119 (8), 2379–2387. doi: 10.1172/JCI38813 19620772 PMC2719936

[B30] Qian L. Weng X. Chen W. Sun C. Wu J. (2014). TREM-1 as a Potential Therapeutic Target in Neonatal Sepsis. Int. J. Clin. Exp. Med. 7 (7), 1650–1658. 25126161 PMC4132125

[B31] Ruiz-Pacheco J. A. A. Vivanco-Cid H. Izaguirre-Hernández I. Y. Y. Estrada-García I. Arriaga-Pizano L. Chacón-Salinas R.. (2014). TREM-1 Modulation During Early Stages of Dengue Virus Infection. Immunol. Lett. 158 (1–2), 183–188. doi: 10.1016/j.imlet.2014.01.003 24447863

[B32] Samant M. Sahu U. Pandey S. C. Khare P. (2021). Role of Cytokines in Experimental and Human Visceral Leishmaniasis. Front. Cell Infect. Microbiol. 11, 1–18. doi: 10.3389/fcimb.2021.624009 PMC7930837 33680991

[B33] Sharma S. Davis R. E. Srivastva S. Nylén S. Sundar S. Wilson M. E. (2016). A Subset of Neutrophils Expressing Markers of Antigen-Presenting Cells in Human Visceral Leishmaniasis. J. Infect. Dis. 214 (10), 1531–1538. doi: 10.1093/infdis/jiw394 27601622 PMC5091370

[B34] Sonnenberg G. F. Nair M. G. Kirn T. J. Zaph C. Fouser L. A. Artis D. (2010). Pathological Versus Protective Functions of IL-22 in Airway Inflammation are Regulated by IL-17a. J. Exp. Med. 207 (6), 1293–1305. doi: 10.1084/jem.20092054 20498020 PMC2882840

[B35] van Bremen T. Drömann D. Luitjens K. Dodt C. Dalhoff K. Goldmann T.. (2013). Triggering Receptor Expressed on Myeloid Cells – 1 (Trem-1) on Blood Neutrophils is Associated With Cytokine Inducibility in Human E. Coli Sepsis. Diagn. Pathol. 8 (1), 707. doi: 10.1186/1746-1596-8-24 PMC3584978 23414215

[B36] van Zandbergen G. Hermann N. Laufs H. Solbach W. Laskay T. (2002). Leishmania Promastigotes Release a Granulocyte Chemotactic Factor and Induce Interleukin-8 Release But Inhibit Gamma Interferon-Inducible Protein 10 Production by Neutrophil Granulocytes. Infect. Immun. 70 (8), 4177–4184. doi: 10.1128/IAI.70.8.4177-4184.2002 12117926 PMC128123

[B37] Yizengaw E. Getahun M. Tajebe F. Cruz Cervera E. Adem E. Mesfin G.. (2016). Visceral Leishmaniasis Patients Display Altered Composition and Maturity of Neutrophils as Well as Impaired Neutrophil Effector Functions. Front. Immunol. 7, 1–12. doi: 10.3389/fimmu.2016.00517 27965662 PMC5126105

