# Supplementary material for: TREM-1 Expression on the Surface of Neutrophils in Patients With Visceral Leishmaniasis Is Associated With Immunopathogenesis
Source: Front Cell Infect Microbiol. 2022 Mar 24;12:863986. doi: 10.3389/fcimb.2022.863986 (PMC8988227; doi:10.3389/fcimb.2022.863986)
Supplement: Supplementary file 1 [file Table_1.docx]

**Supplementary Table 1**: Correlation between serum levels of IL-22 with inflammatory mediators from patients with VL at different times of leishmanicidal treatment.

|  | IL-22 | |
| --- | --- | --- |
|  | **r** | **P value** |
| TNF-α | 0.5115 | 0.0448 |
| IL-4 | 0.5562 | 0.0222 |
| IL-5 | 0.6589 | 0.005 |
| IL-6 | 0.6618 | 0.0048 |
| IL-12p70 | 0.759 | 0.0006 |
| IFN-γ | 0.4724 | 0.0577 |
| sTREM-1 | 0.233 | 0.3653 |
| IL-17A | 0.9013 | <0.0001 |
